# A rapid method for determining arachidonic:eicosapentaenoic acid ratios in whole blood lipids: correlation with erythrocyte membrane ratios and validation in a large Italian population of various ages and pathologies

**DOI:** 10.1186/1476-511X-9-7

**Published:** 2010-01-27

**Authors:** Angela M Rizzo, Gigliola Montorfano, Manuela Negroni, Laura Adorni, Patrizia Berselli, Paola Corsetto, Klaus Wahle, Bruno Berra

**Affiliations:** 1 Dipartimento di Scienze Molecolari Applicate ai Biosistemi, Università degli Studi di Milano, Italy; 2 School of Medicine and Dentistry, University of Aberdeen, UK

## Abstract

**Background:**

Omega-3 and -6 polyunsaturated fatty acids (LCPUFA), are important for good health conditions. They are present in membrane phospholipids.

The ratio of total n-6:n-3 LCPUFA and arachidonic acid:eicosapentaenoic acid (AA and EPA), should not exceed 5:1. Increased intake of n-6 and decreased consumption of n-3 has resulted in much higher, ca 10/15:1 ratio in RBC fatty acids with the possible appearance of a pathological "scenario". The determination of RBC phospholipid LCPUFA contents and ratios is the method of choice for assessing fatty acid status but it is labour intensive and time consuming.

**Aims of the study:**

[i] To describe and validate a rapid method, suitable for large scale population studies, for total blood fatty acid assay; [ii] to verify a possible correlation between total n-6:n-3 ratio and AA:EPA ratios in RBC phospholipids and in whole-blood total lipids, [iii] to assess usefulness of these ratio as biomarkers of LCPUFA status.

**Methods:**

[1] Healthy volunteers and patients with various pathologies were recruited.

[2] Fatty acid analyses by GC of methyl esters from directly derivatized whole blood total lipids and from RBC phospholipids were performed on fasting blood samples from 1432 subjects categorised according to their age, sex and any existing pathologies.

AA:EPA ratio and the total n-6:n-3 ratio were determined.

**Results:**

AA:EPA ratio is a more sensitive and reliable index for determining changes in total blood fatty acid and it is correlated with the ratio derived from extracted RBC phospholipids.

**Conclusions:**

The described AA:EPA ratio is a simple, rapid and reliable method for determining n-3 fatty acid status.

## Background

The evidence that diet is a key component of general health is well accepted. Governments are developing health strategies that propose to modify the diet and lifestyle of their citizens in order to reduce the incidence of diet-related conditions such as obesity, cardiovascular disease, cancer, type 2 diabetes and mental health problems and reduce the health-associated social and healthcare costs.

Food manufacturers have reacted to the new market demands for healthier products in two ways. Firstly, they have attempted to eliminate or reduce "negative" nutrients, such as *trans*-fats, saturated fat, sugars with high glycaemic index and salt, and secondly, they are adding ingredients to their products with known and substantiated health benefits. An example of the latter approach is the fortification of many foods with the long-chain, omega-3 polyunsaturated fatty acids (omega-3 LCPUFAs), [eicosapentaenoic acid (EPA) and docosohexaenoic acid (DHA)] found naturally in human breast milk, in marine algae and in the oil of fish such as tuna, mackerel, herring, salmon and sardines.

Numerous studies have reported a variety of beneficial effects of omega-3 fatty acids on human health [1].

In humans, EPA (20:5n-3) and DHA (22:6n-3) can be biosynthesized from the parent essential fatty acid alpha-linolenic acid (ALA; 18:3n-3). However, due to low conversion rates of ALA into EPA, and particularly DHA, the dietary intake of ALA must be relatively high and the intake of LA, a metabolic competitor for the biosynthetic pathway, low if this pathway was to meet the body's needs for the longer chain derivatives. Fish do not synthesize the greater proportion of these compounds found in their tissues. They are obtained from single-cell marine organisms that fish and shellfish regularly consume. EPA and DHA are found mainly in fish that live in cold, deep seas [2].

The omega-6 PUFAs can be found in vegetable oils, and they are present in smaller amounts in breast milk. The parent essential fatty acid of the omega-6 family is linoleic acid (C18:2n-6, LA). LA, ALA and their metabolic products, arachidonic acid (C20:4n-6, AA) together with EPA and DHA play a key role as structural and functional components of cellular and intracellular membranes throughout the human body, but especially in brain, heart, retina, and testes.

Many international agencies suggest that LCPUFAs should provide about 7% of total calories and that the omega-6/omega-3 ratio should be no more than 5:1 [3]. Recent reports suggest that the total dietary amounts of these fatty acids are also important and that diets with similar ratios of n-6:n-3 elicit different metabolic effects depending on their actual amounts. Increasing absolute amounts of α-linolenic acid whilst keeping the ratios of n-6:n-3 the same resulted in a greater conversion of the precursor fatty acids to EPA and DHA which highlighted the competitive interactions of these substrates for the elongation/desaturation/membrane incorporation pathways in man. The amounts of other fatty acids in membrane lipids would reduce the availability of the AA and EPA after phospholipolysis and would be expected to alter EFA (Essential Fatty Acids) metabolism. This does not preclude the usefulness of the AA:EPA ratio in membrane and whole blood phospholipids as a biomarker for omega-6:omega-3 status in man.

AA is released from phospholipids by phospholipase A2 and is the precursor of the eicosanoids, which include prostaglandins of the 2 series (PGE_2_, PGD_2_), leukotrienes of the 4 series (LTA_4_, LTB_4_, LTC_4_, LTD_4_, LTE_4_), and lipoxines [4]. Their production is catalyzed by cyclooxygenase, lipooxygenase and epoxygenase enzymes respectively. These omega-6 derived eicosanoids have numerous, physiologically important roles including augmenting inflammation, modulating immunity and promoting platelet aggregation and vasoconstriction.

The omega-3 EPA can compete with AA for the same enzymes to form different classes of eicosanoids, namely 3 series PGs and 5 series LTs which can counteract the deleterious effects of 2-series prostanoids.

Omega-6 and omega-3 fatty acids are both incorporated into membrane phospholipids when the AA/EPA ratio is between 1:1 to 5-10:1. When the ratio is higher, the incorporation of AA is preferred, suggesting a greater affinity of the enzymes for EPA [5]. The predicted production of the 2-series eicosanoids therefore increases, giving rise to pro-inflammatory and pro-aggregatory conditions.

The omega-3 LCPUFAs present a wide range of beneficial properties. Studies *in vitro *and with animal models have indicated that they affect membrane lipid composition, blood lipid profiles, eicosanoids biosynthesis, cell signalling cascades, gene expression and the functioning of the cardiovascular system; they are involved in ameliorating or preventing the aetiology of a number of different pathologies [6-9].

It is now recognized that omega-3 LCPUFAs have an important role in growth and development of the nervous system and retina and are involved in the regulation of cognitive and visual functions and in general mental health [10-12]. The highest concentrations of DHA are found in the cerebral cortex, synaptic vesicles and synaptosomes [13-16]. DHA represents the prevalent fatty acid in cerebral gray matter phospholipids, comprising 45% to 65% of the total fatty acid content in phosphatidylserine of the CNS [7].

An increasing number of reports demonstrate that DHA-containing phospholipids can influence membrane properties such as permeability, fusion, and plane elasticity [6].

The effect of omega-3 LCPUFAs on gene expression is shown by their influence on the plasma triglyceride (TG) profile. The only fully recognized therapeutic action of omega-3 is the clinically certified decrease of plasma TG concentration following their supplementation [17-19] which is due to the up-regulation of enzymes involved in fatty acid β-oxidation and down-regulation of enzymes of fatty acid synthesis [20].

Another positive effect of omega-3 LCPUFAs is the control of cardiac arrhythmia that is linked to the modulation of specific calcium ion channels in cardiomocytes [21,22].

Many observational studies (e.g. in cancer and neurodegenerative diseases) have postulated the possible therapeutic potential of omega-3 LCPUFAs supplementation, due to the observation that plasma and tissues of the affected subjects showed a low content of omega-3 LCPUFAs, in particular of EPA [23-28]. However, the usefulness of the clinico-chemical methods and indices used to assess the LCPUFA content in the cells and blood of subjects are labour-intensive and difficult to compare. Total omega-6 versus total omega-3 fatty acid ratios in different circulating cells and in blood plasma has been used, but comparisons with the more specific AA/EPA ratio are few and show some discrepancies.

The primary aim of this study was to evaluate the AA/EPA ratio in directly derivatised whole blood as a simple metabolic index of the LCPUFA status of subjects of different ages, omega-3 intake and pathologies by comparing it with the total omega-6/omega-3 fatty acid ratios in whole blood and in RBC membrane phospholipids, which has historically been the standard for assessing longer-term intake of the LCPUFA in man. We analysed whole blood samples from subjects classified by age, sex and health status, with or without self-motivated omega-3 supplement consumption, using a simple, direct derivatisation method. RBC cell membranes were isolated, their phospholipids were extracted by standard chromatographic procedures, their fatty acids derivatised and analysed by gas chromatography to assess specific n-6:n-3 ratios in cell membrane phospholipids by the standard method.

## Materials and methods

### Subjects

Fasted blood samples from 1432 subjects, who were referred by their physicians, were analysed to assess their AA/EPA and total n-6:n-3 ratios in whole blood and in RBC membrane phospholipids. The subjects declared their age, sex and health status, and willingness to receive an omega-3 supplement. The dose of the supplement, when used, was 2-3 g/day of EPA and DHA in a ratio of 2:1.

As reported in Table 1, 300 apparently healthy subjects did not consume omega-3, while 344 consumed omega-3 regularly. Out of a total of 798 subjects suffering from various pathological diseases, 577 did not consume any supplement and 211 consumed a supplement on a regular basis.

**Table 1:** The fatty acid profiles (% composition) of whole blood lipids in subjects from an Italian population not consuming omega-3 supplements and arranged according to age

**%**	**Under 20 ** **years (15-20)**		**21-40 ** **years**		**41-60 ** **years**		**Over 60 ** **years (61-81)**			
**n**	**19**		**103**		**127**		**51**			
		
	**Mean**	**S.E.**	**Mean**	**S.E.**	**Mean**	**S.E.**	**Mean**	**S.E.**		
C16:0 *	21.634	0.535	22.226	0.295	22.779	0.194	23.441	0.343		
C16:1**	1.252	0.228	1.392	0.066	1.438	0.064	1.945	0.140		
**C18:0**	12.321	0.547	11.197	0.259	11.364	0.170	10.836	0.251		
C18:1**	22.154	0.823	23.959	0.385	23.537	0.330	25.728	0.600		
C18:2n-6** (LA)	23.089	0.735	22.155	0.347	21.795	0.371	19.527	0.463		
**C18:3n-3 (ALA)**	0.431	0.036	0.451	0.028	0.450	0.023	0.477	0.030		
**C20:3n-6**	2.098	0.117	1.932	0.056	1.860	0.060	2.174	0.289		
C20:4n-6* (AA)	12.145	0.454	11.412	0.200	11.064	0.171	10.690	0.280		
**C20:5 n-3 (EPA)**	0.670	0.093	1.022	0.111	1.132	0.077	1.040	0.090		
**C22:5n-3 (DPA)**	1.381	0.106	1.264	0.048	1.266	0.044	1.203	0.060		
**C22:6n-3 (DHA)**	2.825	0.293	2.989	0.100	3.315	0.115	2.940	0.150		
tot ω-6**	37.332	0.845	35.499	0.403	34.719	0.381	32.391	0.624		
**tot ω-3**	5.307	0.394	5.727	0.197	6.163	0.198	5.659	0.240		

a) Overall significance by ANOVA: * *p *< 0.05; ** *p *< 0.01.

To determine any further correlation, the healthy subjects were grouped by age, while pathological subjects were grouped by declared disease. Numbers in each disease category and in healthy individual groups are given in the graphs.

### Methods

Blood (2 ml) was drawn from an antecubital vein and maintained at 2-4°C for no more than 24 hours from the time of sampling to methylester preparation.

An aliquot of whole blood was derivatised directly, as described below, for GC analysis of the fatty acid composition.

Erythrocytes (RBC) were separated by centrifugation (2000 rpm for 10 minutes, ALC 4232 centrifuge) and stored at -70°C in isotonic buffer until used for membrane purification.

Cell membranes of RBC (ghosts) were prepared by lysis with hypotonic buffer (phosphate 5 mM, pH 8, EDTA 0.5 mM), precipitated by centrifugation and washed several times to eliminate haemoglobin residues.

Ghost lipids were extracted with chloroform/methanol according to Folch [29], and fractionated by silicic acid chromatography (200-400 mesh BIORAD, CA) into non-polar lipids, glycolipids and phospholipids [30].

### GC fatty acid analysis

The fatty acid composition of whole blood and of RBC membrane phospholipids was determined as follows. RBC membrane phospholipids were isolated, extracted and separated by standard procedures prior to derivatisation to their component fatty acid methyl esters (FAME) by sodium methoxide. FAME of whole blood were obtained by direct derivatization of an aliquot of freshly drawn blood with sodium methoxide in methanol 3.33% (w/v). FAME were rapidly extracted into petroleum ether/diethyl ether and rapidly dried. They were then injected into a gas chromatograph (Agilent Technologies 6850 series II, CA) equipped with flame ionization detector. (Capillary column, AT Silar length 30 m, film thickness 0.25 μm; carrier gas, helium; injector temperature, 250°C; detector temperature, 275°C). The oven temperature was controlled at 50°C for 2 min and then increased at a rate of 10°C min^-1 ^to 200°C, and maintained there for 20 min. A standard mixture containing all fatty acid methylesters (Sigma-Aldrich, MO) was injected for calibration each day [31].

Total omega-6/omega-3 fatty acids and the AA/EPA ratios were calculated for whole blood and for RBC membrane phospholipids.

### Statistical analysis

Results were expressed as mean ± SEM; all the data were analyzed by analysis of variance (ANOVA). Student's *t*-test was used for comparisons between two groups.

## Results

**Figure 1:**
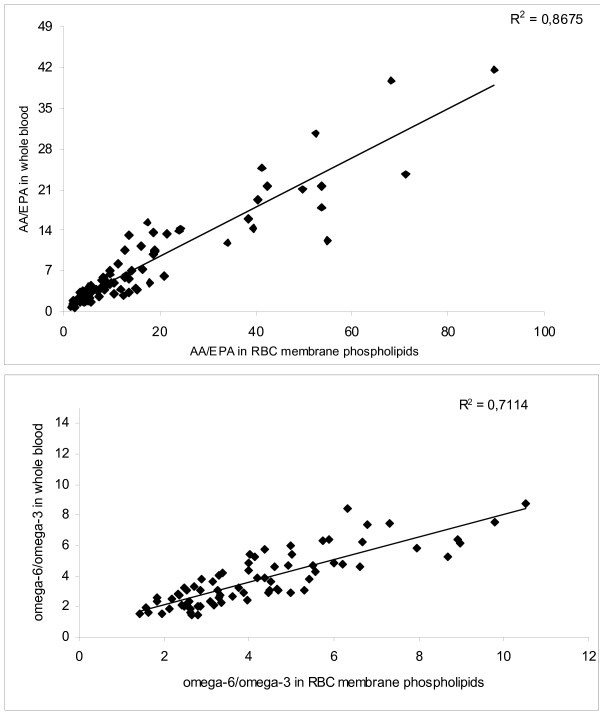
**Comparison of the linear correlations between the total omega-6/omega-3 ratios (A) and the AA/EPA ratios (B) in whole blood and in RBC membrane**.

A comparison between the total omega-6:omega-3 ratios in directly derivatised whole blood and those in isolated RBC membrane phospholipids from 150 patients showed a good correlation (R^2 ^= 0.711) (Figure 1a). However, the comparison of the more specific AA/EPA ratios in whole blood and in RBC membrane phospholipids showed a much stronger correlation (R^2 ^= 0.868) (Figure 1b).

Healthy subjects, that did not take any fish oil supplements, were divided into four groups according to age: [i] under 20 years, [ii] 21-40 years, [iii] 41-60 years, and [iv] over 60 years. The fatty acid profiles (% composition) of whole blood lipids in the four age groups are reported in Table 1. Many differences in fatty acid proportions become apparent, such as a decrease of palmitic acid with an increase of palmitoleic and oleic acids, with increasing age of the subjects. No difference were related to sex (data not shown).

**Figure 2:**
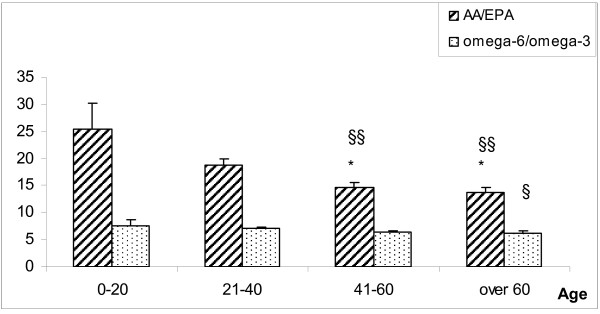
**The AA/EPA and total omega-6/omega-3 ratios in whole blood of healthy subjects not using fish oil supplements and grouped by age**. Mean ± S.E; Student's *t*-test: * *p *< 0.05 vs 0-20; §* p *< 0.05 vs 21-40; §§ *p *< 0.01 vs 21-40, number of subjects as in table 1.

Figure 2 depicts the AA/EPA ratios and total omega-6/omega-3 ratios in the whole blood of the four groups of healthy subjects who did not consume omega-3 supplements and were classified by age as in Table 1. There was an overall decrease of the AA/EPA ratio with aging, with statistical significance observed only for subjects older than 40 years.

This difference resulted from a slight increase of EPA with a significant decrease of AA (Table 1). Also, there was a slight increase in DHA content from under 20 years up to 60 years of age but a marked decrease for subjects over 60 years old. Smaller differences were observed in the total omega-6/omega-3 ratio, related to age, even when there was a significant decrease of total omega-6 fatty acids (Table 1).

**Figure 3:**
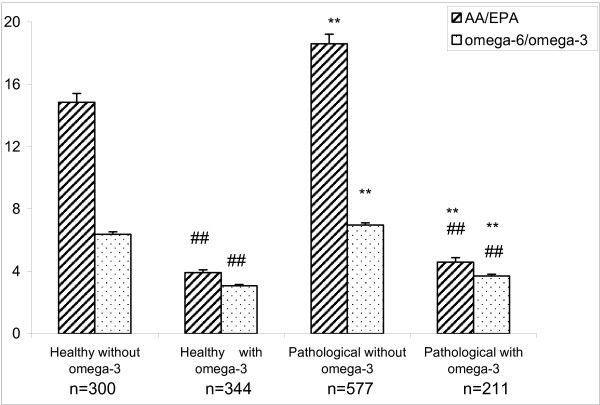
**AA/EPA and omega-6/omega-3 ratios in whole blood of healthy subjects and in a group of patients with various pathologies, with and without consumption of omega-3**. Mean ± S.E; Student's *t*-test: ## *p *< 0.01 with omega-3 vs without omega-3; ** *p *< 0.01 pathological vs healthy; number of subjects as in Table 1.

The total omega-6/omega-3 and AA:EPA ratios in the whole blood of healthy subjects and in patients with various diseases, with or without omega-3 supplementation, are compared in Figure 3. The AA:EPA ratio in healthy subjects who did not consume omega-3 supplements was high relative to values recommended by nutritional guidelines. In healthy subjects consuming omega-3, the ratio, as expected, was significantly lower, but with values in a range that is considered to be safe.

Patients suffering from various diseases, irrespective of the type of pathology and the consumption of omega-3, had higher AA:EPA ratios than healthy subjects suggesting a decreased omega-3 intake or increased turnover, or an increased omega-6 intake, or decreased turnover. Nevertheless, in both patients with disease and in healthy subjects, the voluntary consumption of an omega-3 supplement (2-3 g of EPA/DHA) resulted in a statistically significant decrease of the AA:EPA ratio indicating that the methodology was very sensitive to such changes in fatty acid intake. The total omega-6:omega-3 ratio was also higher in subjects with disease compared to healthy subjects, although the differences were less marked compared to those of the AA:EPA ratio.

**Figure 4:**
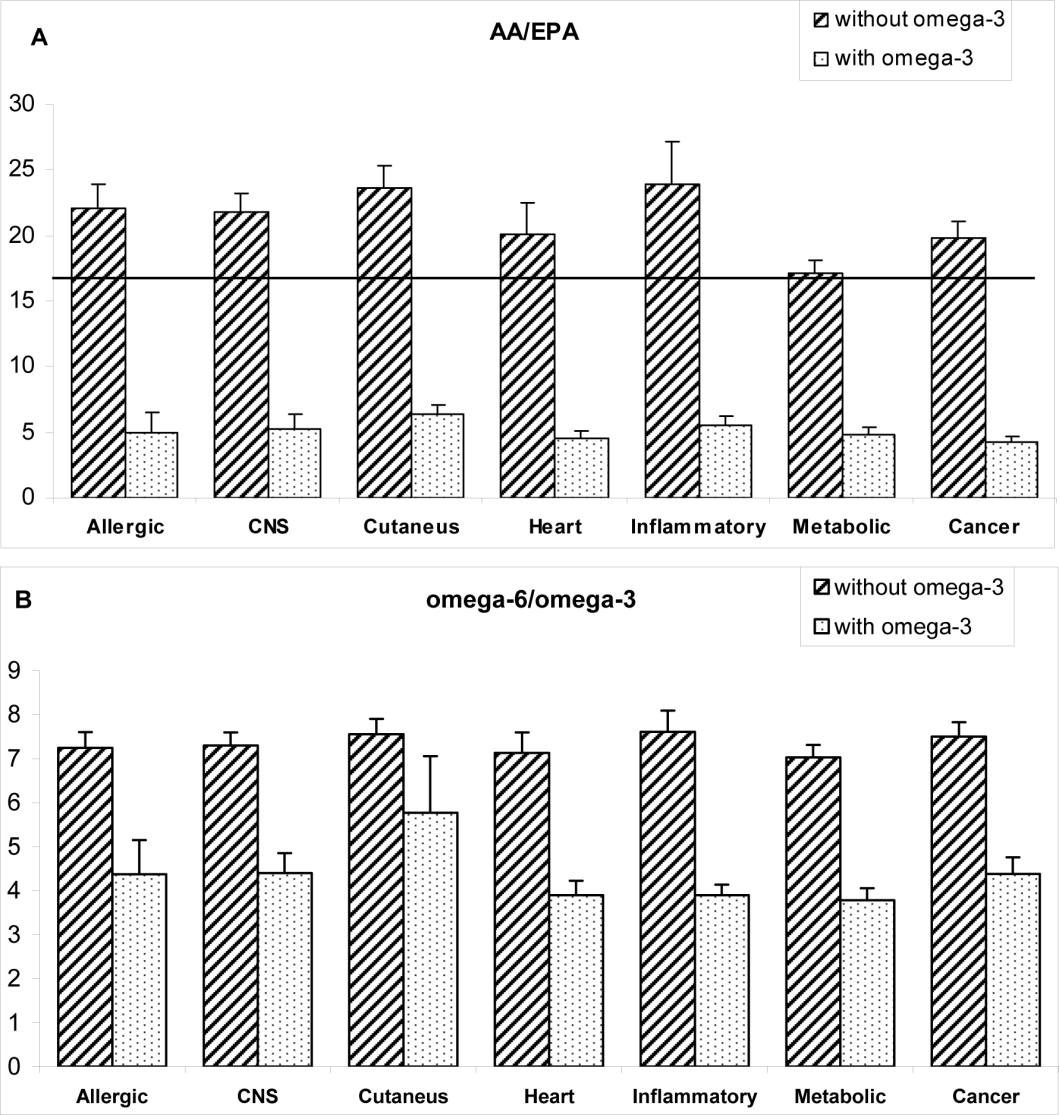
**(A) AA/EPA ratios and (B) omega-6/omega-3 ratios in whole blood of patients grouped according to their specific pathologies**. The horizontal lines indicate the mean value for all the healthy subjects that do not use omega-3.

AA:EPA and total omega-6:omega-3 ratios in directly derivatised whole blood from patients with different pathologies are shown in Figure 4A and 4B, respectively. The number of subjects in each disease category is shown on the graph. The upper horizontal lines indicate the mean values found in healthy subjects not taking omega-3 supplements. Subjects that did not take omega-3 supplements and suffered from allergic, neurodegenerative, skin and inflammatory diseases had higher values for AA:EPA ratios than those with the other diseases (heart, metabolic, cancer). Ratios in all the pathologies were, however, reduced in patients that used an omega-3 supplement, albeit to slightly different degrees. From the data in Figure 4B, less marked differences were observed for each disease when the total omega-6:omega-3 ratio of membrane/blood lipids was determined.

## Discussion

The essential LCPUFAs play important and positive roles in maintaining normal physiological conditions and consequently health in humans. Some chronic pathologies are associated with defective metabolism of these fatty acids, particularly the omega-6 linoleic and arachidonic acids and the omega-3 eicosapentaenoic (EPA) and docosahexaenoic (DHA) LCPUFAs.

Analysis of the composition of these fatty acids in phospholipids of RBC membranes provides information about their longer-term (approximately 125 days, RBC lifespan) dietary intake and their biosynthetic conversion to eicosanoids and certain metabolic anomalies (e.g. delta 6-desaturase deficiency). In clinical practice, determination of RBC membrane phospholipid fatty acid composition is a standard diagnostic procedure for evaluating essential fatty acid status and for use as a biomarker for different pathologies [32,33], particularly concerning cardiovascular diseases [34-36]. However, assessing this biomarker requires a lengthy, labour intensive, multi-step analytical procedure.

We have tested a rapid, simple and reliable method to determine AA:EPA and total omega-6:omega-3 ratios in whole blood lipids against the standard RBC membrane phospholipid composition method. The AA:EPA ratios in the directly derived whole blood lipids were very closely correlated with those obtained by the standard method from RBC membrane phospholipids in a large population group. The more specific AA:EPA ratios also showed better correlations than the total omega-6:omega-3 ratios using both whole blood and membrane methods (R^2 ^= 0.868 vs 0.711 respectively). This suggested that the former was the more sensitive ratio to use. The method was validated in healthy subjects of varying ages and in patients with various pathologies. A statistically significant decrease of the AA:EPA ratio was observed in subjects over 40 years old, which resulted from a simultaneous increase of EPA and a decrease of AA, irrespective of gender. Less significant differences were observed for the total omega-6:omega-3 ratio, even when the total content of omega-6 was decreased significantly. Previous studies on a Canadian population showed a higher level of omega-3 only in elderly subjects [37], probably due to an increased consumption of seafood, although a decreased oxidation of omega-3 in elderly compared to younger volunteers may also play a role [38]. As a working hypothesis and according to observations on prostaglandin synthesis [39], we suggest that the lower level of circulating AA may also be attributed, at least in part, to a reduction of Δ6 desaturase activity in elderly subjects. However, these observations need to be confirmed by larger studies.

The AA:EPA ratio in healthy Italian subjects without supplementation is high in the present study and is comparable to that reported for other Western populations where the ratio of omega-6 to omega-3 fatty acids in the diet is also high [40,41]. In subjects using an omega-3 supplement, the ratios were significantly decreased but the values were considered to be safe and more beneficial to health [9].

Patients with allergic, skin and neurodegenerative diseases had higher ratios of AA:EPA compared to the values for subjects with other pathologies. The reasons for this are not immediately apparent but could reflect differences in intake and/or metabolism, particularly an increased turnover of EPA. More work is required with individuals presenting with different pathologies and consuming varying levels of omega-3 before the observed ratios can be considered as actual biomarkers for specific diseases.

Similar results to those with the AA:EPA ratios were obtained for the total omega-6:omega-3 ratios in both whole blood and RBC membrane phospholipids, although in most cases the differences were much smaller, probably due to the diet of the subjects that may include varying amounts of omega-6 fatty acids, particularly linoleic acid from vegetable oils.

In conclusion, our observations show that a simple, direct derivatisation method of assessing fatty acid profiles in whole blood is a rapid, reliable and reproducible tool for determining the fatty acid status of subjects of different ages and pathologies. The simplicity of the method makes it particularly useful for assessing the fatty acid status of large cohorts where the use of RBC membrane phospholipids would be a daunting prospect.

## Competing interests

The authors declare that they have no competing interests.

## Authors' contributions

AMR conceived and designed the study and performed analysis and interpretation of data, GM performed lipid analysis and statistical analysis, MN performed lipid analysis, LA performed lipid analysis, PB performed lipid analysis, PC performed lipid analysis, KW has been involved in drafting the manuscript and revising it critically for important intellectual content, BB Coordinated the study and participated in its design.

All authors have read and approved the final manuscript.
